# Functional Features of Alginates Recovered from *Himanthalia elongata* Using Subcritical Water Extraction

**DOI:** 10.3390/molecules26164726

**Published:** 2021-08-04

**Authors:** Noelia Flórez-Fernández, Herminia Domínguez, María Dolores Torres

**Affiliations:** Department of Chemical Engineering, Faculty of Sciences, University of Vigo, Edificio Politécnico, As Lagoas s/n, 32004 Ourense, Spain; noelia.florez@uvigo.es (N.F.-F.); matorres@uvigo.es (M.D.T.)

**Keywords:** autohydrolysis, biopolymer, brown seaweed, antitumoral, rheology

## Abstract

Subcritical water extraction of *Himanthalia elongata* and the subsequent acetone fractionation to precipitate crude fucoidans generated a liquid phase which was used to recover alginates with a wide range of viscoelastic features and other soluble extracts with potential biological activities. The precipitated alginate was converted to sodium alginate using an environmentally friendly treatment before being characterized by Fourier transform infrared attenuated total reflectance, nuclear magnetic resonance, high performance size exclusion chromatography and rheological measurements. The cell viability of three human cell lines (A549, HCT-116, T98G) in the presence of the extracts obtained before and after acetone fractionation was assessed. Fractionation with different acetone volumes showed a slight effect in the behavior of the different tested cell lines. Results also indicated a notable effect of the processing conditions on the block structure and molar mass of the extracted biopolymer, with the subsequent impact on the rheological properties of the corresponding gelled matrices.

## 1. Introduction

Seaweeds have been explored in the last decades. The biological properties associated with their compounds have been responsible for this increased interest [1]. The cell wall of the seaweeds contains, mainly, polysaccharides (fucoidan, alginate, agar, carrageenan, agarose and others) [2]. The colloids of the algae have a broad range of applications in different industries [3] such as food, pharmaceutic and cosmeceutical [4,5,6,7]. Furthermore, other bioactive compounds are present in the cell wall of the algae. In this context, development of an extraction strategy to obtain these compounds is necessary. Different technologies have been used to achieve this challenge, especially those focusing on eco-friendly processes using water as a solvent, a concept fitting in a sustainability approach [8]. Further fractionation steps as an acetone treatment modify the dielectric constant of water favoring the precipitation of sulfated polysaccharides, with the consequent impact on their structure and the corresponding functional properties [9].

Macroalgae metabolites have an important role because some of them can display positive effects on human health [10]. Anti-inflammatory, antitumoral, anticoagulant, antioxidant, anti-obesity and other biological properties have been associated with these macroalgae metabolites in recent research [5,11].

Asian countries have algae as part of their diet, and more recently, the consumption in other territories is increasing based on nutritional and biological properties. The edible brown seaweed *Himanthalia elongata*, widely known as “sea spaghetti”, belongs to the order of Fucales [12]. As occurs with other brown seaweeds, composition is directly influenced by abiotic factors, and also the extraction process can impact the biological properties. Moreover, brown seaweeds have other polysaccharide with potential biological activities, mainly fucoidan and alginate [13,14,15]. Fucoidans are mostly comprised of fucose and sulfate groups, and other components, such as glucose, mannose, xylose and rhamnose are also present. The content and location of the sulfate groups affect the bioactive properties, which are highly dependent on the previous extraction and fractionation treatments [16]. 

**Figure 1:**
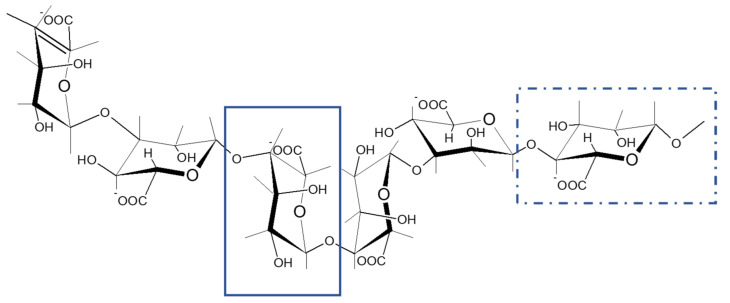
Structure of a possible configuration of alginate molecule. Guluronic acid is represented by a continuous line and mannuronic acid is represented by a dashed line.

Alginate is a biopolymer with suitable properties, such as biocompatibility, biodegradability and low toxicity, to be used for biomedical applications such as wound healing, drug delivery, tissue repair and regeneration [7,17,18]. This biopolymer is only present in brown seaweeds, providing algae flexibility and stability against marine currents. Furthermore, other properties are also associated with this biopolymer such as thickener, stabilizer or gelling agent with potential application in the industry. Alginate is comprised of mannuronic and guluronic acid units (Figure 1). According to their proportion, alginate can feature different molecular mass distributions and rheological properties in terms of viscosity, elasticity or thermal stability, which is critically relevant in order to define the potential final applications [15,19]. 

Different studies have reported the biological activities of alginate as prebiotic, antitumor, anti-diabetic, anti-hypertensive, antimicrobial, immunomodulatory, anticoagulant or antioxidant, among others [15,20]. The antitumoral features of alginate have been reported from the point of view of the prevention of tumor cell proliferation. Nowadays, chemotherapy is a widely used treatment in cancer treatment, but some drugs (such as cisplatin) have limited use due to toxicity issues. In this context, marine sources (such as macroalgae) could have suitable properties to be used in biomedical applications [21]. 

The retrieving of high-value biomolecules from *H. elongata* using subcritical water extraction was previously proposed [22], but the influence of the process on the sodium alginate fraction was not assessed. The current work is focused on the structural and rheological characterization of sodium alginate obtained from this edible brown seaweed after subcritical water and acetone fractionation, and provides further insight on the biological potential of the recovered soluble extracts.

## 2. Results and Discussion

### 2.1. General Overview

One of the components of the brown seaweed is fucoidan, which is associated with different biological activities. Previous work by the authors of this study was focused on the extraction of this polysaccharide as well as assessment of its antioxidant and the cytotoxicity activities [22]. Furthermore, the behavior of the calcium alginate from the liquid phase of subcritical water extraction obtained at 160 °C was studied. It should be indicated that extracts recovered after hydrothermal process at the selected temperature were labelled as SWE, whereas those obtained after acetone fractionation were labelled as (SWE_A). Steady-shear flow curves and frequency sweeps of the dispersions prepared with the biopolymer extracted from the liquor of *H. elongata* were evaluated. The outcomes exhibited a similar behavior when compared with the commercial alginate, promoting an integral use of the edible *H. elongata* brown seaweed.

**Figure 2:**
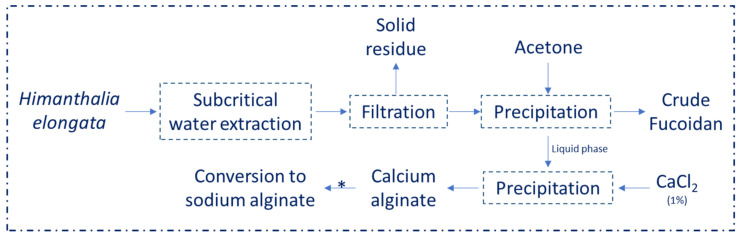
General overview of *H. elongata* extraction processing to the recovery of sodium alginate fraction. * The process to convert calcium in sodium alginate was previously reported in [26].

By analyzing the composition of *H. elongata* used here as raw material and reported in a previous work of these authors [22], it becomes evident that this edible alga is in consonance with others works where similar results were obtained [23,24]. Nevertheless, differences could be due to collection season, geographical location and abiotic factors [25]. Even then, brown seaweeds have some special biopolymers in their composition, such as alginate and fucoidan, which are both only present in brown algae, and both with an attractive potential in biomedical applications [14]. In this context, in the actual work metabolic activity was evaluated for the extracts obtained by SWE and, also, for the extracts obtained by acetone fractionation, increasing the behavior of the samples from *H. elongata* in several cell lines. Moreover, calcium alginate was converted to sodium alginate using a procedure previously reported [26], making this new biopolymer the main focus of the structural and rheological studies developed here (Figure 2).

### 2.2. Antiproliferative Action of Extracts

Table 1 collects the study of the cell viability of the extracts obtained from *H. elongata* in tested human tumoral cell lines: epithelial lung adenocarcinoma (A549), colon carcinoma (HCT-116) and Caucasian human glioblastoma (T98G). The lowest cell viability was shown for the extract obtained at 160 °C using a concentration of 25 µg/mL with a value of approximately 44%. In the previous work [22], lung (NCI-H460), ovarian (A2780) and breast (MCF-7) cell lines were assessed at 160 °C and 220 °C, the temperatures selected based on the maximum sulfate and phlorotannins content, respectively. It was reported that the cell inhibition was higher for lung and breast cell lines for the extract obtained at 220 °C. For the ovarian cell line, a slight increase of the inhibition was observed for the extract obtained at 160 °C [22]. Taking into account the new data obtained in the present work, the observed behaviors could be partially explained by the fucose:sulfate ratio. 

The cell viability of the extracts obtained after fractionation with different acetone volumes were evaluated, and different behavior was observed. The same concentration (500 µg/mL) and volume extraction (0.5 v) showed two distinct values: 30.5% and close to 57% of cell viability for the cell lines HCT-116 and T98G, respectively. These tendencies can be related to the differences of the cell lines. A549 cell line, for the same acetone fractionation volume, at the concentration of 500 µg/mL led to a cell viability of 47.5%, when the precipitation was performed with 1.5 v. 

### 2.3. Alginate

The yield of sodium alginate recovered from liquid phases obtained during SWE treatment of *H. elongata* was around 5.9%. The magnitude of this parameter increased with increasing the volume ratios of acetone:hydrolyzate from 0.5 to 2.0 *v*/*v*, achieving values of 15.9%. No notable differences were identified when 2.5 volumes of acetone were employed. This alginate extraction yield is in the range of that achieved for different alginophyte species using conventional extraction treatments, varying from around 3% to 40%, such as *Sargassum muticum* (3%), *Sargassum turbinarioides* (10%), *Sargassum asperifolium* (12%), *Fucus guiryi* (13.6%), *Fucus vesiculosus* (16.2%), *Laminaria ochroleuca* (27.5%) or *Lessonia nigrescens* (34–41%) [27]. It should be noted that the alginate content determined here is still higher than that previously found from other Fucales species (10%) [28].

#### 2.3.1. Structural Properties

**Figure 3:**
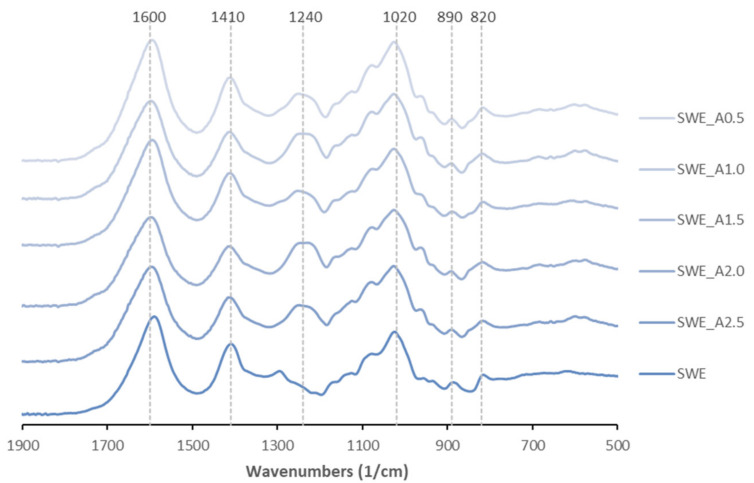
FTIR-ATR profiles of sodium alginate extracted from *H. elongata* after subcritical water extraction (up to 160 °C) followed by acetone fractionation (SWE_A0.5–SWE_A2.5) using 0.5–2.5 acetone:liquid extract volume ratio, *v*/*v*.

Figure 3 presents the FTIR-ATR spectra of above sodium alginates obtained after acetone fractionation (from SWE_A0.5 to SWE_A2.5). Note here that the numbers after SWE_A correspond with the acetone volume ratio used for the fractionation. The spectrum of the sodium alginate recovered after subcritical water extraction at 160 °C (SWE) is also displayed for comparative purposes. Similar profiles were identified in all cases, independently of the acetone ratio used. The major intensity signals, found at wavenumbers around 1600 1/cm, were attributed to O–C–O and C=O asymmetric stretching vibrations of uronic acids; the bands at 1410 1/cm corresponded to C–OH deformation vibration [29]. The peaks at 1240 1/cm, attributed to the existence of sulfate groups, were stronger for alginates recovered after acetone fractionation (SWE_A0.5–SWE_A2.5). The signals around 1020 1/cm, related to the C-O polysaccharides vibrations, were of intermediate intensity for all tested sodium alginates. Bands of minor intensity identified at 890 1/cm were assigned to the α-l-guluronic asymmetric ring vibration, and at 820 1/cm to the β-mannuronic acid. These spectra are consistent with those previously reported for extracts from *H. elongata* using conventional Soxhlet extraction [30]. It should be remarked that FTIR-ATR spectra of alginates from previous studies of other Fucales species extracted with different methods (water, acid, enzymatic extraction) did not show relevant differences either [31].

Table 2 summarizes the compositional block structural features of the above sodium alginates obtained from ^1^HNMR spectra. The well-known characteristic signals identified in the spectra (i.e., guluronic acid anomeric proton (about 5.2 ppm), guluronic acid (about 4.6 ppm), mannuronic acid anomeric proton (about 4.8 ppm) and the alternating blocks (about 4.9 ppm) together with the corresponding areas were employed for the quantitative assessment of the guluronic and mannuronic acids (F_G_, F_M_), M/G ratios and diad frequencies (F_GG_, F_MM_, F_GM_, F_MG_) [32].

Acetone fractionation involved a clear impact on F_M_, F_G_ and the corresponding M/G ratios of tested sodium alginates. Not only was a clear increase of F_G_ with acetone increase observed, but also a significant drop of F_M_ and M/G. The magnitude of this latter parameter indicated higher values of mannuronic than guluronic acid blocks. This behavior (M/G > 1) is consistent with the results previously reported for alginates from *Himanthalia elongata* [33] as well as other Fucales species, such as *Sargassum muticum* (1.04), *Sargassum vulgare* (1.27) or *Fucus vesiculosus* (1.44–1.84) [27]. It should be remarked that M/G ratio magnitude can provide relevant insight to define the alginate final application. Those biopolymers with lower G content usually provide lower viscosity and higher flexibility, being attractive to develop nanoparticles or polyelectrolyte complexes, whereas those with low M content usually feature higher viscosity, being adequate to formulate gelled matrices for food and non-food applications [26]. However, it is necessary to take into account not only the effect of the homopolymeric block structures, but also the alternating blocks. Intermediate F_MM_ as well as low F_GG_ and alternating blocks (F_MG_ = F_GM_) favored the gelling capability of these alginates [28]. Overall, SWE and the subsequent acetone fractionation had a remarked impact on the alginate structure and in their mechanical potential.

**Figure 4:**
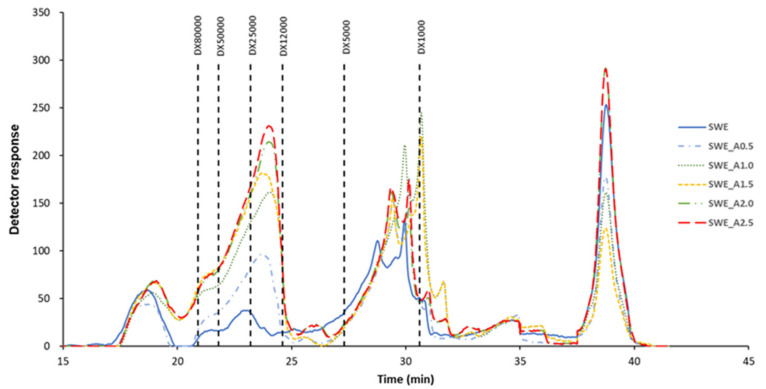
HPSEC profiles of sodium alginate extracted from *H. elongata* after representative subcritical water extraction (160 °C) followed by acetone fractionation (0.5–2.5 acetone:liquid extract volume ratio, *v*/*v*).

Figure 4 shows the corresponding HPSEC profiles of the sodium alginates recovered from *H. elongata* after acetone fractionation. An increase in the depolymerization of the sodium alginate was observed with increasing acetone content up to SWE_A2.0, without notable differences with SWE_A2.5. The major peaks corresponding to the highest molecular mass displayed a progressive increase with acetone ratio, between 12,000–25,000 g/mol. The compounds with the molecular weight (around 1000 g/mol) increased with acetone ratio 1.0v and 1.5v and decreased at 2.0v and 2.5v. It was observed that both, increase and decrease, allowed similar levels as in the original extract (SWE). The figure shows a clear impact on the molecular weight distribution related to the acetone ratio used. A relevant effect on this parameter of other biopolymers as fucoidans was previously reported for the acetone fractionation of the liquid phases at the same temperature gathered from *H. elongata* [22]. The latter work showed that the highest fucoidan fraction of the liquid phases was found at about 5 kDa, and was shifted to 25 kDa in the presence of the highest acetone values. It was also observed that sodium alginates with the average lowest molecular mass presented the highest M/G ratios. In this context, the areas of the peaks corresponding with dextrans > 12,000 g/mol follow the trend acetone ratio 2.5 > 2.0 > 1.5 > 1.0 > 0.5, which is consistent with the M/G ratios behavior, consistently with the outcomes previously reported for other Fucales species [34]. A certain degree of depolymerization of sodium alginate has been found after other purification treatments that could be taken advantage of to obtain fluids with lower viscosity values [35]. Despite that high molecular mass alginates can exhibit stronger gelling features, a distribution with low molecular masses is necessary to control the high viscosity properties favoring the industrial processing.

#### 2.3.2. Thermo-Rheological Properties

**Figure 5:**
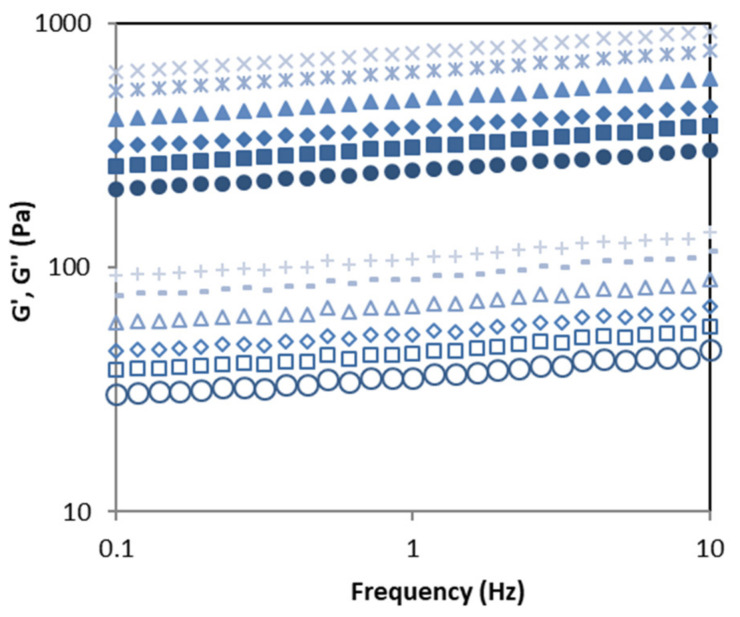
Frequency sweeps of gelled matrices made with sodium alginate extracted from *H. elongata* after representative subcritical water extraction (160 °C) followed by acetone fractionation (0.5–2.5 acetone:hydrolyzed, *v*/*v*). Symbols: G′, closed symbols; G″, open symbols, circles (SWE), squares (SWE_A0.5), diamonds (SWE_A1.0), triangles (SWE_A1.5), double blades (G′, SWE_A2.0), blades (G′, SWE_A2.5), crosses (G″, SWE_A0.5), lines (G″, SWE_A0.5).

Figure 5 displays the effect of acetone fractionation on the thermo-rheological properties of matrices formulated with the corresponding sodium alginates made in CaCl_2_ at 20 °C. A characteristic gel behavior can be observed for all matrices (i.e., G′ > G″, both moduli almost frequency independent). Note here that G′ exhibited values around 10-times larger than G″ over the tested frequency range. A clear impact of the acetone fractionation of tested sodium alginates was identified in the developed gelled matrices, since both moduli increased at a fixed frequency with acetone increasing up to SWE_A2.0. Again, no notable differences were noticed for SWE_A2.5. These tendencies are consistent with those previously described for alginate structural blocks and molecular mass distributions as sodium alginates with the highest molecular mass as well as the lowest M content and consequently M/G ratios (SWE_A2.0, SWE_A2.5) led to the development of the gels with the highest viscoelastic features. The low magnitude of F_MG_ = F_GM_ blocks also promoted the gel formation, as previously reported for other Fucales species [26]. According to the G′ and G″ moduli values, intermediate gels strengths were obtained in all cases. Despite this fact, all gelled matrices exhibited viscoelastic magnitudes within those found in commercial foodstuff as gelling desserts [36].

**Figure 6:**
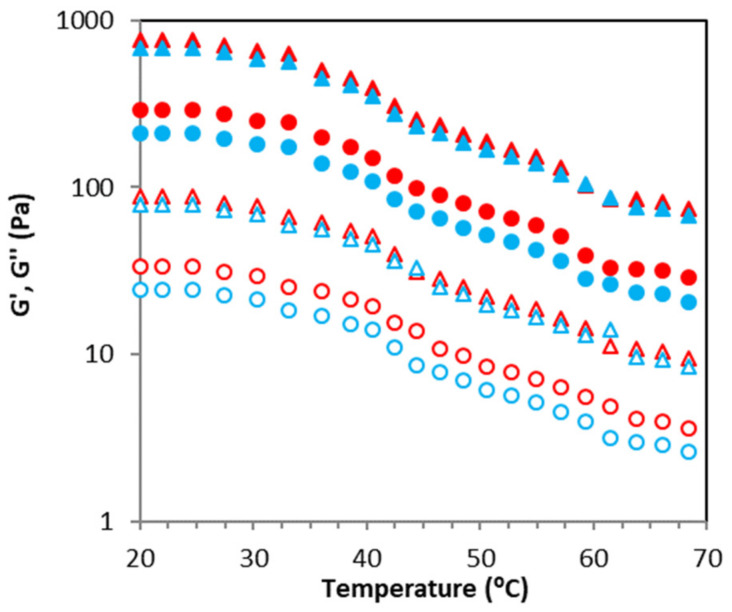
Representative thermal heating (red symbols) and cooling (blue symbols) profiles of tested systems. Symbols: G′, closed symbols; G″, open symbols, circles (SWE), triangles (SWE_A2.0).

The influence of acetone fractionation on the thermal features of recovered sodium alginates is presented in Figure 6. Temperature ramps of gelled matrices showed predominant elastic behavior (about 10-times). In heating ramps, G′ and G″ moduli decreased with increasing temperature over the tested temperature range. While assessing the behavior at a fixed temperature, it was observed that both viscoelastic moduli increased with increasing acetone content used during the fractionation step. Similar tendencies were observed for the corresponding cooling sweeps. Note here that a quick stability step was identified for tested alginates (<2.5 min) determined in the performed time sweeps, which is an indication of the mechanical stability of tested alginates with the consequent industrial relevance. These results agree with those previously reported for gelled matrices prepared with ultrasound treated alginates from other Fucales species [26]. No gelling/melting temperatures were identified in the tested cooling/heating assays within the studied temperature range. The frequency sweeps performed after thermal analysis did not show notable differences with those presented in Figure 5, suggesting strong thermo-reversible features of the studied sodium alginate. Finally, it should also be remarked that no water syneresis was identified for gelled matrices formulated with sodium alginates recovered after acetone fractionation of SWE liquid phases of *H. elongata* for two weeks of cold storage.

## 3. Materials and Methods

### 3.1. Raw Materials

Dehydrated *Himanthalia elongata* was purchased to Algas Atlanticas Algamar S.L. (Pontevedra, Spain). The industrial processing of these seaweeds comprised a fresh cleaning stage, followed by drying at 42 °C in a convective oven. After reception, the seaweeds were ground and stored in darkness at room temperature in opaque plastic bags until further use.

### 3.2. Extraction Treatment

Subcritical water extraction (SWE) was performed under non isothermal conditions up to the maximal heating temperature to obtain soluble extracts from the brown seaweed *H. elongata*, according to a previous work [22]. Briefly, the extraction treatment was performed in a pressurized reactor with continuous stirring (Parr Instruments series 4842, USA) using a ratio of 1:30 (*w*/*w*) alga:water, the temperature increase up to the selected temperature (160 °C) and then the reactor was quickly cooled down to room temperature. Liquid and solid phases were separated by vacuum filtration.

Acetone fractionation was used to precipitate oligosaccharides from the liquid phase, and the acetone:liquid extract volume ratios were observed to influence the recovery yields [22]. Briefly, the volume ratios were increased from 0.5 to 2.0 (*v*/*v*), mixed and kept at 4 °C for 24 h. Then, they were separated by centrifugation for further analysis. 

### 3.3. Alginate Fraction

Alginate was precipitated with CaCl_2_ (1% *w*/*w*) from the liquid fractions obtained after acetone fractionation. The appropriate amount of the above salt was added to the liquid phase and stirred for 4 h before overnight cold storage. Then, in order to separate the calcium alginate, systems were centrifuged (15 min, 4000 rpm). According to a greener approach for the conversion to sodium alginate comprehensively detailed in a previous work [26], the calcium biopolymer was carefully treated. Briefly, hydrochloric acid commonly used in the well-known conventional procedure was substituted by lemon juice (up to pH 3); and sodium carbonate was added to get the alginic acid sodium salt. Afterwards, this biopolymer was dried in a vacuum oven (−0.8 bar, 40 °C, 48 h).

#### 3.3.1. Fourier Transform Infrared Attenuated Total Reflectance (FTIR-ATR)

FTIR-ATR tests of the above dried sodium alginates (2 mg) were conducted on a Nicolet 6700 (Thermo Scientific Infrared Spectrophotometer, USA) equipped with a deuterated-triglycine sulfate detector (DGTS). Samples were blended with KBr (10 mg), pressed at 7 ton and dried using an infrared lamp (30 min). FTIR-ATR spectra were recorded (400–4000 nm, 32 scan/min) using the OPUS-2.52 software (Opus Software Limited, Grantham, UK).

#### 3.3.2. Proton Nuclear Magnetic Resonance (^1^H NMR)

^1^H NMR spectra of above sodium alginates were monitored on a Bruker ARX400 (Bruker BioSpin GmbH spectrometer, Steuerberater, Germany). Measurements of the biopolymers solutions (10 mg/mL) were made using deuterated water as a solvent, 3-(trimethylsilyl)-l-propane sulfonic acid (Sigma-Aldrich, St. Louis, MO, USA) as an internal standard, and operated at 400 MHz and 75 °C. Note here that ^1^H NMR signals corresponding to the anomeric protons of the mannuronic (M) and guluronic (G) acids were reported elsewhere at 4.70 and 5.08 ppm, respectively. Moreover, the M/G ratio was calculated according to the procedure previously described [26].

#### 3.3.3. High Performance Size Exclusion Chromatography (HPSEC)

HPSEC chromatograms of tested alginates and SWE were performed in a 1260 series Hewlett-Packard chromatograph (Agilent, Waldbornn, Germany) with a refractive index detector. The sample was injected with autosampler and the volume was 5 µL. Two columns 300 × 7.8 mm (TSKGel G2500PW_XL_ and TSKGel G3000PW_XL_, Tosoh Bioscience, Zürich, Germany) in series and one 40 × 6 mm PWX-guard column were used as measuring systems. Measurements were run using Milli Q water (0.4 mL/min) as mobile phase and dextrans (1000–80,000 g/mol) (Fluka, St. Louis, MO, USA) as calibration standards. The corresponding molar mass distribution chromatograms were recorded using the ChemStation for LC systems software (Agilent Technology, Germany).

#### 3.3.4. Rheology

Sodium alginate was dispersed in distilled water at a usually employed biopolymer content (1.0 g/L) and room temperature. It should be remarked that CaCl_2_ (0.1 mol/L) was used as a gelling agent according to the method reported elsewhere [26].

Monitoring of the viscoelastic behavior of the sodium alginate gelled matrices was carried out through small amplitude oscillatory shear testing in a MCR302 rheometer (Anton Paar, Austria, Germany). Gelled biopolymer based systems were placed on the sand blasted plate-plate measuring system (1 mm gap, 25 mm diameter). Light paraffin oil was employed to seal the sample edges in order to prevent water release during rheological testing. All systems were rested for 10 min to allow thermal and structural equilibration prior to the experiments. Firstly, stress sweeps (20 and 70 °C, 1 Hz) were performed to define the linear viscoelastic region (<72 Pa) for the alginate gelled matrices. Then, the rheology study consisted of a 6-step procedure: (1) frequency sweep (20 °C, 20 Pa) to define the viscoelastic gel characteristics (i.e., elastic, G′, and viscous, G″, moduli), (2) heating thermal sweep up to 70 °C (1 °C/min, 20 Pa, 1 Hz), (3) time sweep (70 °C, 15 min, 20 Pa, 1 Hz), (4) cooling thermal sweep to 20 °C (1 °C/min, 20 Pa, 1 Hz), (5) time sweep (20 °C, 15 min, 20 Pa, 1 Hz) and (6) frequency sweeps (same conditions as in step 1). Steps 2 to 6 allow evaluation of the thermal stability of the sodium alginates.

#### 3.3.5. Syneresis of the Gelled Matrices

The water syneresis of the above gelled matrices was also evaluated following the experimental method described in detail by [37]. Briefly, gels were stored in centrifuge tubes in the fridge for two weeks. Afterwards, gelled matrices were centrifuged at 2000 g for 20 min, and the syneresis estimated as the percentage between the water release and the weight of the gelled matrices.

### 3.4. Antitumoral Features of the Extracts

The cell viability of the extracts obtained by subcritical water extraction and, after acetone fractionation, was evaluated for three human cell lines: epithelial lung adenocarcinoma (A549), colon carcinoma (HCT-116) and Caucasian human glioblastoma (T98G). All of them were provided by the European Collection of Cell Culture (ECCC) and were tested by the Thiazolyl Blue Tetrazolium Bromide (MTT, Sigma) method [19]. The extracts of *H. elongata* and those obtained after acetone fractionation were tested at concentrations below 500 µg/mL, Stauosporine (Biomar collection, AQUAe, Mérida, Spain) was used as a positive control, with IC_50_ values against A549 cells (0.001 µg/mL), PSN1 cells (0.001 µg/mL), HCT-116 cells (0.005 µg/mL), and T98G cells (0.001 µg/mL), and as negative controls, the specific medium for each cell line without cells and untreated cells was used.

### 3.5. Statistical Analysis

All above experiments were performed at least in triplicate. Above data were assessed using one-factor analysis of variance, ANOVA. A post-hoc Scheffé test was performed to differentiate means with 95% confidence (*p* < 0.05) using the PASW Statistics v.22 software (IBM SPSS Statistics, New York, NY, USA).

## 4. Conclusions

To conclude, it should be indicated that acetone fractionation of sodium alginates recovered after subcritical water extraction of *H. elongata* is an adequate method to obtain a wide range of biopolymers with suitable extraction yields, different molecular mass distributions and block structures. The proposed processing conditions allow developing gelled matrices of intermediate strength with short maturation times, without jeopardizing thermal and viscoelastic stability. The absence of syneresis is another advantage of formulated gelled matrices. A slight impact of the extraction conditions on the cellular viability of tested epithelial lung adenocarcinoma, colon carcinoma and Caucasian human glioblastoma cell lines was observed.

## Figures and Tables

**Table 1:** Influence on the cell viability of the extraction process on the extract obtained at 160 °C (SWE) and the acetone fractionation volumes (SWE_A) tested in three cell lines: A549 (epithelial lung adenocarcinoma), HCT-116 (colon carcinoma) and T98G (Caucasian human glioblastoma). Data represent mean (n ≥ 3) ± SEM.

	Cell Viability (%)		
Cell Line	A549	HCT-116	T98G
SWE	43.71 ± 3.22 (25 µg/mL)	50.86 ± 1.02 (25 µg/mL)	48.85 ± 2.37 (25 µg/mL)
SWE_A	47.56 ± 5.68 (500 µg/mL)—1.5 v	30.50 ± 4.70 (500 µg/mL)—0.5 v	56.87 ± 3.34 (500 µg/mL)—0.5 v

**Table 2:** Impact of the acetone fractionation on the sodium alginate fractions extracted from *H. elongata*.

Alginate	M/G	F_M_	F_G_	F_MM_	F_GG_	F_MG_ = F_GM_
SWE	1.94 ^a^	0.66 ^a^	0.34 ^e^	0.58 ^a^	0.26 ^a^	0.08 ^e^
SWE_A0.5	1.78 ^b^	0.64 ^b^	0.36 ^d^	0.54 ^b^	0.26 ^a^	0.10 ^d^
SWE_A1.0	1.56 ^c^	0.61 ^c^	0.39 ^c^	0.48 ^c^	0.26 ^a^	0.13 ^c^
SWE_A1.5	1.44 ^d^	0.59 ^d^	0.41 ^b^	0.44 ^d^	0.26 ^a^	0.15 ^b^
SWE_A2.0	1.27 ^e^	0.56 ^e^	0.44 ^a^	0.38 ^e^	0.26 ^a^	0.18 ^a^
SWE_A2.5	1.27 ^e^	0.56 ^e^	0.44 ^a^	0.38 ^e^	0.26 ^a^	0.18 ^a^

In all cases, standard deviations were <0.01. Data values in a column with different superscript letters are significantly different at the *p* ≤ 0.05 level.

## Data Availability

Data is contained within the article.

## References

[1] White W.L., Wilson P. (2015). World Seaweed Utilization. Seaweed Sustainability.

[2] Stiger-Pouvreau V., Bourgougnon N., Deslandes E., Fleurence J., Levine I. (2016). Chapter 8—Carbohydrates from Seaweeds. Seaweed in Health and Disease Prevention.

[3] Bixler H.J., Porse H. (2011). A decade of change in the seaweed hydrocolloids industry. J. Appl. Phycol..

[4] Couteau C., Coiffard L. (2016). Seaweed Application in Cosmetics. Seaweed in Health and Disease Prevention.

[5] Kartik A., Akhil D., Lakshmi D., Panchamoorthy Gopinath K., Arun J., Sivaramakrishnan R., Pugazhendhi A. (2021). A critical review on production of biopolymers from algae biomass and their applications. Bioresour. Technol..

[6] Fleurence J. (2016). Seaweeds as Food. Seaweed in Health and Disease Prevention.

[7] Zhang H., Cheng J., Ao Q. (2021). Preparation of alginate-based biomaterials and their applications in biomedicine. Mar. Drugs.

[8] Mahadevan K. (2015). Seaweeds: A Sustainable Food Source.

[9] Acevedo-García V., Flórez-Fernández N., López-García M., Vilariño J.M.L., Domínguez H., Torres M.D. (2021). Acetone Precipitation of Heterofucoidans from *Sargassum muticum* Autohydrolysis Extracts. Waste Biomass Valoriz..

[10] Déléris P., Nazih H., Bard J.M. (2016). Seaweeds in Human Health. Seaweed in Health and Disease Prevention.

[11] Gómez-Zorita S., González-Arceo M., Trepiana J., Eseberri I., Fernández-Quintela A., Milton-Laskibar I., Aguirre L., González M., Portillo M.P. (2020). Anti-obesity effects of macroalgae. Nutrients.

[12] Garcia-Vaquero M., Lopez-Alonso M., Hayes M. (2017). Assessment of the functional properties of protein extracted from the brown seaweed *Himanthalia elongata* (Linnaeus) S. F. Gray. Food Res. Int..

[13] Torres M.D., Flórez-Fernández N., Simón-Vázquez R., Giménez-Abián J.F., Díaz J.F., González-Fernández A., Domínguez H. (2020). Fucoidans: The importance of processing on their anti-tumoral properties. Algal Res..

[14] Zhu B., Ni F., Xiong Q., Yao Z. (2021). Marine oligosaccharides originated from seaweeds: Source, preparation, structure, physiological activity and applications. Crit. Rev. Food Sci. Nutr..

[15] Liu J., Yang S., Li X., Yan Q., Reaney M.J.T., Jiang Z. (2019). Alginate oligosaccharides: Production, biological activities, and potential applications. Compr. Rev. Food Sci. Food Saf..

[16] Ale M.T., Meyer A.S. (2013). Fucoidans from brown seaweeds: An update on structures, extraction techniques and use of enzymes as tools for structural elucidation. RSC Adv..

[17] Reig-Vano B., Tylkowski B., Montané X., Giamberini M. (2021). Alginate-based hydrogels for cancer therapy and research. Int. J. Biol. Macromol..

[18] Ahmad Raus R., Wan Nawawi W.M.F., Nasaruddin R.R. (2021). Alginate and alginate composites for biomedical applications. Asian J. Pharm. Sci..

[19] Flórez-Fernández N., Torres M.D., González-Muñoz M.J., Domínguez H. (2019). Recovery of bioactive and gelling extracts from edible brown seaweed *Laminaria ochroleuca* by non-isothermal autohydrolysis. Food Chem..

[20] Torres M.D., Kraan S., Domínguez H. (2019). Seaweed biorefinery. Rev. Environ. Sci. Biotechnol..

[21] Xing M., Qi C., Yu W., Han X., Jiarui Z., Qing Z., Aiguo J., Shuliang S. (2020). Advances in research on the bioactivity of alginate oligosaccharides. Mar. Drugs.

[22] Cernadas H., Flórez-Fernández N., González-Muñoz M.J., Domínguez H., Torres M.D. (2019). Retrieving of high-value biomolecules from edible *Himanthalia elongata* brown seaweed using hydrothermal processing. Food Bioprod. Process..

[23] Gómez-Ordóñez E., Alonso E., Rupérez P. (2010). A simple ion chromatography method for inorganic anion analysis in edible seaweeds. Talanta.

[24] Gómez-Ordóñez E., Jiménez-Escrig A., Rupérez P. (2010). Dietary fibre and physicochemical properties of several edible seaweeds from the northwestern Spanish coast. Food Res. Int..

[25] Endo H., Suehiro K., Gao X., Agatsuma Y. (2017). Interactive effects of elevated summer temperature, nutrient availability, and irradiance on growth and chemical compositions of juvenile kelp, *Eisenia bicyclis*. Phycol. Res..

[26] Flórez-Fernández N., Domínguez H., Torres M.D. (2019). A green approach for alginate extraction from Sargassum muticum brown seaweed using ultrasound-assisted technique. Int. J. Biol. Macromol..

[27] Belattmania Z., Kaidi S., El Atouani S., Katif C., Bentiss F., Jama C., Reani A., Sabour B., Vasconcelos V. (2020). Isolation and FTIR-ATR and 1H NMR characterization of alginates from the main alginophyte species of the atlantic coast of Morocco. Molecules.

[28] Fenoradosoa T.A., Ali G., Delattre C., Laroche C., Petit E., Wadouachi A., Michaud P. (2010). Extraction and characterization of an alginate from the brown seaweed *Sargassum turbinarioides* Grunow. J. Appl. Phycol..

[29] Gómez-Ordóñez E., Rupérez P. (2011). FTIR-ATR spectroscopy as a tool for polysaccharide identification in edible brown and red seaweeds. Food Hydrocoll..

[30] Mateos-Aparicio I., Martera G., Goñi I., Villanueva-Suárez M.J., Redondo-Cuenca A. (2018). Chemical structure and molecular weight influence the in vitro fermentability of polysaccharide extracts from the edible seaweeds *Himathalia elongata* and *Gigartina pistillata*. Food Hydrocoll..

[31] Borazjani N.J., Tabarsa M., You S.G., Rezaei M. (2017). Effects of extraction methods on molecular characteristics, antioxidant properties and immunomodulation of alginates from *Sargassum angustifolium*. Int. J. Biol. Macromol..

[32] Fertah M., Belfkira A., Dahmane E.m., Taourirte M., Brouillette F. (2017). Extraction and characterization of sodium alginate from Moroccan *Laminaria digitata* brown seaweed. Arab. J. Chem..

[33] Pereira L., Gheda S.F., Ribeiro-Claro P.J.A. (2013). Analysis by vibrational spectroscopy of seaweed polysaccharides with potential use in food, pharmaceutical, and cosmetic industries. Int. J. Carbohydr. Chem..

[34] Fawzy M.A., Gomaa M., Hifney A.F., Abdel-Gawad K.M. (2017). Optimization of alginate alkaline extraction technology from *Sargassum latifolium* and its potential antioxidant and emulsifying properties. Carbohydr. Polym..

[35] Sari-Chmayssem N., Taha S., Mawlawi H., Guégan J.P., Jeftić J., Benvegnu T. (2016). Extracted and depolymerized alginates from brown algae *Sargassum vulgare* of Lebanese origin: Chemical, rheological, and antioxidant properties. J. Appl. Phycol..

[36] Torres M.D., Raymundo A., Sousa I. (2013). Effect of sucrose, stevia and xylitol on rheological properties of gels from blends of chestnut and rice flours. Carbohydr. Polym..

[37] García-Ríos V., Ríos-Leal E., Robledo D., Freile-Pelegrin Y. (2012). Polysaccharides composition from tropical brown seaweeds. Phycol. Res..

